# Fullerene-Based Symmetry in *Hibiscus rosa-sinensis* Pollen

**DOI:** 10.1371/journal.pone.0102123

**Published:** 2014-07-08

**Authors:** Kleber Andrade, Sara Guerra, Alexis Debut

**Affiliations:** Centro de Nanociencia y Nanotecnología, Universidad de las Fuerzas Armadas ESPE, Sangolquí, Ecuador; University of Zurich, Switzerland

## Abstract

The fullerene molecule belongs to the so-called super materials. The compound is interesting due to its spherical configuration where atoms occupy positions forming a mechanically stable structure. We first demonstrate that pollen of *Hibiscus rosa-sinensis* has a strong symmetry regarding the distribution of its spines over the spherical grain. These spines form spherical hexagons and pentagons. The distance between atoms in fullerene is explained applying principles of flat, spherical, and spatial geometry, based on Euclid’s “Elements” book, as well as logic algorithms. Measurements of the pollen grain take into account that the true spine lengths, and consequently the real distances between them, are measured to the periphery of each grain. Algorithms are developed to recover the spatial effects lost in 2D photos. There is a clear correspondence between the position of atoms in the fullerene molecule and the position of spines in the pollen grain. In the fullerene the separation gives the idea of equal length bonds which implies perfectly distributed electron clouds while in the pollen grain we suggest that the spines being equally spaced carry an electrical charge originating in forces involved in the pollination process.

## Introduction

The Rose of China, *Hibiscus rosa-sinensis*, belongs to the family Malvaceae. There exist about 200 forms of the genus Hibiscus [1]. The species commonly called Chinese rose is a shrubby plant, probably native to tropical regions of Asia, and is widely used as ornamental species. Usually, pollination in plants is a process with many variables that together form a delicate balance ensuring their survival. Pollen plays a large biological role in plant perpetuation. It must be organized in a certain way in order to overcome all natural barriers for its transference from the anther to the final destination, the stigma. Considering that a large percentage of the human nutrition has its origin in plants, an understanding of pollination is critical to preserve the food chain [2]. Numerous researches have been dedicated to pollen morphology since its structure is fundamental for the identification of species [3], [4]. Pollination is indeed a process clearly explained by physics and biology, nevertheless the field is still under intense research [5], [6]. There are few reports on Rose of China pollen, and they mainly focus on the definition of qualitative structural parameters [7] as a way to identify the species of Hibiscus, without being conclusive. The aim of this paper is to characterize the morphology of *Hibiscus rosa-sinensis* pollen and to provide an outline of relevant and measurable structural parameters described both analytically and geometrically. In particular, we will demonstrate that some of the features are clearly related to modern material structure, fullerene, which opens a new relationship of Palynology with other sciences.

## Materials and Methods

### A. Age group definition

**Figure 1:**
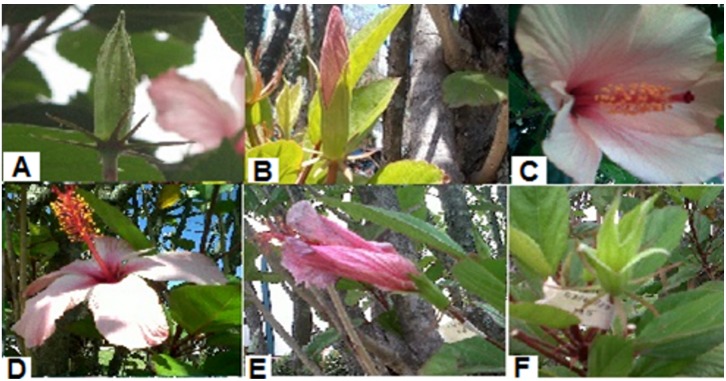
Developing phases of *Hibiscus rosa-sinensis* flower. A: immature stage, pollen is forming and reproductive organs of the flower are not observed, 1–3 days. B: bud stage, the flower enters in pre-anthesis, 4–5 days. C: anthesis stage, 5–8 days. D: final phase of anthesis, pollen remains viable, 9–12 days. E: senescence phase, flower begins to close, 13–15 days. F: the flower separates from the tree.

Fifty flowers of *Hibiscus rosa-sinensis* were sampled at an early immature stage from different trees. Average development time of *Hibiscus rosa-sinensis* was around 15 days, therefore three arbitrary observation times were chosen (i.e., young, adult, old) corresponding to: budding, anthesis and senescence; see Figure 1. For each group, thirty anthers of five specimens were collected for pollen analysis.

### B. Scanning electron microscopy preparation

Pollen was fixed with a 3% glutaraldehyde solution for one hour. A post-fixed treatment was done with 1% osmium tetroxide solution for one hour. Then samples were dehydrated with series of ethanol solutions ranging from 50% to 99%, with a 10% gradient, for a period of 30 minutes each. The samples were lyophilized using an iLShinBiobase freeze dryer, then placed on stubs supports and finally covered with an approximately 20 nm thick gold layer, using a sputter coating Quorum Q150R-ES. Photos were taken using the secondary electron detector, and digital acquisition was done with an ADDA II device from Soft Imaging System. The study was conducted using a scanning electron microscope ZEISS DSM-960A at the Centro de Nanociencia y Nanotecnología of the Universidad de las Fuerzas Armadas ESPE.

### C. Mathematical parameters

**Figure 2:**
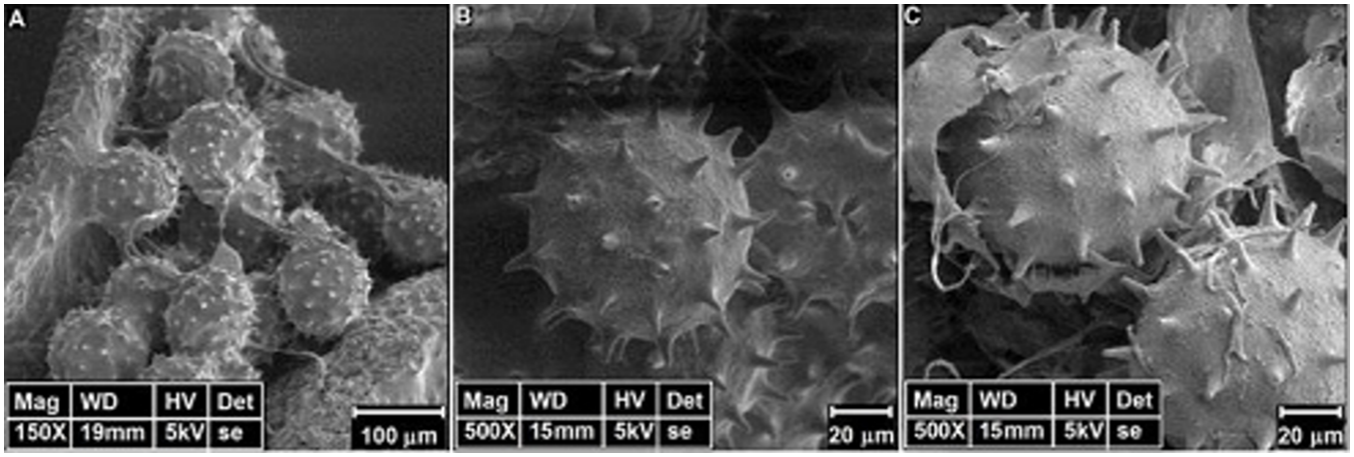
Stages of pollen. A: young stage, pollen grains maintain connections with the anther and the spines are not yet well defined, although they begin to distribute symmetrically over the pollen grains surface. B: adult stage, the pollen has a spherical shape with sharp and long spines. C: old stage, the grain begins to deform, it becomes more vulnerable to treatment for SEM. We identify also that the exine in old stage begins to separate from the surface of pollen, as clearly seen in the picture.

**Figure 3:**
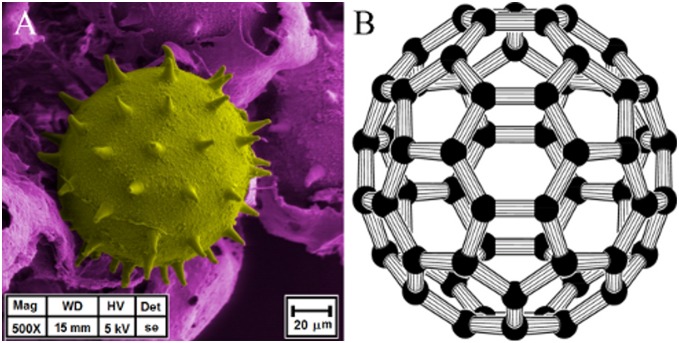
Comparison between an Hibiscus rosa-sinensis grain pollen (A) and a buckminster C60-fullerene molecule (B) (Creative Common License – Rob Hooft). As seen, both structures present a defined distribution of their elements over the whole surface forming hexagons and pentagons.

The classical structure of *Hibiscus rosa-sinensis* pollen is represented in Figure 2. At first glance a symmetrical distribution of spines is clearly identified. In such apparent symmetry, mathematical parameters have been measured and calculated to establish metric relationships of spine distribution over the whole surface of the pollen grain. To do this, a geometrical approach was considered. The buckminster C60-fullerene molecule, Figure 3, has its own symmetric pattern of atom distribution. These two structures, pollen and buckminster C60-fullerene, have the same distribution patterns of their elements over the spherical surface - forming hexagons and pentagons. The only difference is that in the pollen grain each polygon is centered. These features confer to the pollen grains the same classification as a buckminster C60-fullerene molecule regarding the existing symmetry operations.

## Results and Discussion

### A. Tessellation of *Hibiscus rosa-sinensis* grain pollen

The position of pollen spines shown by SEM is similar to those of carbons in the buckminster C60-fullerene molecule. The hexagonal and pentagonal patterns are alike in both structures, giving a specific spherical shape tessellation. According to Euler’s theorem on convex polyhedra, this spherical tessellation with regular spherical hexagons is possible as long as the number of spherical pentagons included is 12. The angle between spines and its similitude with the angle between atoms positions in such a fullerene molecule is a key indication of the symmetry for both structures. Since, to the best of our knowledge, there is no specialized software to measure accurately the spherical shape, spine lengths and pollen angles, we measure these features on the 2D periphery of the spherical grain since they would show actual lengths.

### B. Determination of the angular aperture of two atoms in a buckminster C60-fullerene

The C60 molecule is highly symmetric. Three kinds of rotation axes can be defined. The 5-fold axes through the centers of two facing pentagons, giving a rotation angle of 360/5 = 72 degrees. The rotation axes through two facing, this symmetry is 3-fold, it takes a rotation of 120 degrees to map the molecule onto itself. Finally the 2 fold axis through the centers of the edges, between two hexagons. Since there are 12 pentagons and 20 hexagons, this leads to 120 different symmetry operations. All these forms the icosahedral group which is the point group with the largest number of symmetry operations [8].

**Figure 4:**
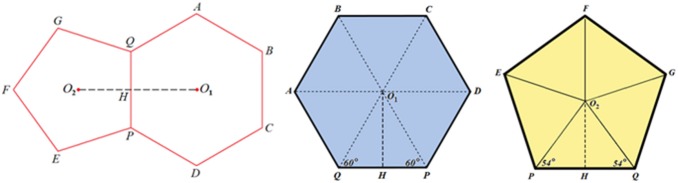
Graphical relationship between a hexagon and a pentagon that share an edge. From this it is possible to calculate what should be the distance between their respective center and vertices (i.e. *O*_1_ *Q*, *O*_1_ *A*, *O*_2_ *P*, *O*_2_ *E*,…) and the magnitude of their respective apothems *O*_1_ *H* and *O*_2_ *H*.



From Figure 4, assuming that the common edge for both the pentagon and hexagon has an arbitrary length of magnitude *a*, the triangle in the hexagon with common edge *PQ* is equilateral with sides equal to *a*. Hence, the apothem *O*_1_ *H* has a length 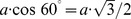. With the pentagon, the triangles similarly formed are not equilateral but isosceles with congruent angles equal to 54^o^ at the vertices that lie on the common edge *PQ*. This gives for each triangle in the pentagon equal sides with a magnitude:
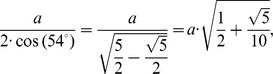
with the apothem *O*_2_ *H* equal to:

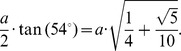



**Figure 5:**
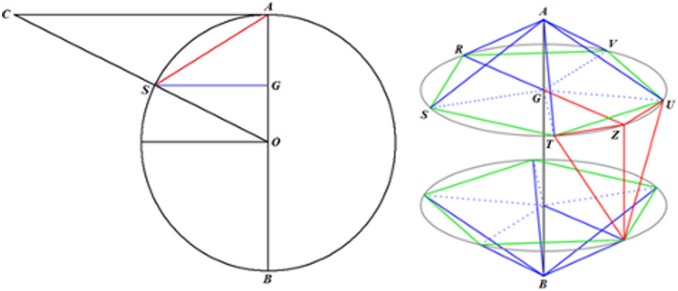
Euclid’s construction showing the length of the corresponding icosahedron inscribed inside the sphere. To the right is a sketch of the main lines that form the top and bottom of the icosahedron.

**Figure 6:**
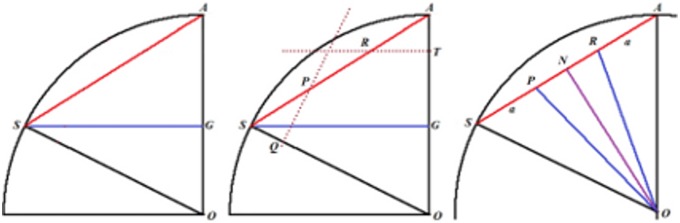
Planes that cut icosahedron edges are perpendicular to the radii subtended to their respective vertices. The edge of the icosahedron is then cut into three segments, the middle one *PR* is the length edge of the truncated icosahedron or fullerene molecule.

The buckminster C60-fullerene molecule is a truncated icosahedron, where its vertices are cut off by planes so that small pentagonal based pyramids are separated from each vertex. Those cut pyramids determine the formation of pentagons and hexagons sharing an edge. Euclid (300 BC) in his book “Elements” describes the icosahedron construction and determines the metric relationship with its containing sphere. In the circle of diameter *AB*, see Figure 5, a perpendicular *AC* is drawn with the same length as the diameter. The line *CO* intersects the circle at point *S.* The segment *AS* is the edge of the icosahedron inscribed within the sphere of diameter *AB*. The metric relationship between the edge length of the icosahedron and unitary radius of the sphere that contains it is:
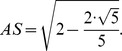



Hence the *AG* side length is:
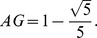



Hence, the trigonometric functions of the angle *ASG* are:
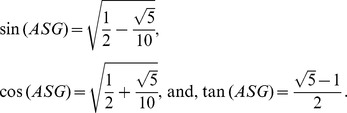



Figure 6 shows the planes cutting vertices and taking off the 3D pentagonal pyramidal volumes, this forms pentagons and hexagons with an edge in common, see Figure 4. The planes that cut the edge *AS* of the icosahedron are planes perpendicular to the radii *AO* and *SO*. When cutting, they determine the formation of two of twelve pentagons, see Figure 5, whose vertices are connected by a full edge *RP* with length *a* and being a common edge of the hexagons Also, it represents the edge for the whole structure of the buckminster C60-fullerene molecule. The centers of formed pentagons lay on the lines *AO* and *SO*, so that segments *PQ* and *RT* are sides of the triangles inside the pentagon whose length depending on the quantity *a* is (Figure 4):
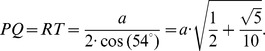



With the length *PQ* it is possible to calculate the length *SP* ( = *AR*) since the angle *ASG* is congruent with the angle *ART,* therefore, see Figure 6:
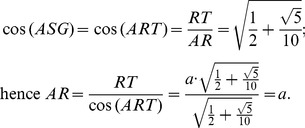



Moreover, the planes that cut the edge length of the icosahedron divide it into three equal lengths; each of them is equal to *a*, so that the edge length of the described fullerene is:
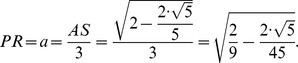



Our goal is to calculate the angular distance subtended by the segment *PR* = *a* on the unit radius sphere containing this structure. The angular distance is the angle *POR*, located in the *POR* isosceles triangle and containing the third part of the icosahedron edge. The height *ON* can be calculated from the *NOA* square triangle:
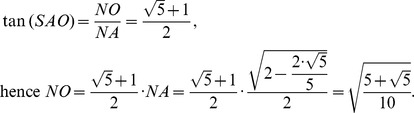



From the isosceles triangle *OPR*, the length of the *PO* = *RO* segments are given by:
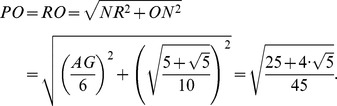



Hence the trigonometric functions of the *POR* angle are:
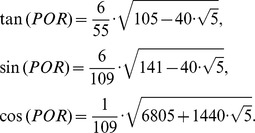



Finally the measure of the *POR* angle is 23.28°, and it is the angle subtended by each of the triangle sides inside the hexagonal figures of the molecule. The isosceles triangles in the pentagon have the angular measurements equal to 20.08°, see Figure 4. So these are the angles that must be found in the pollen structure due to the location of the spines.

### C. Analysis of angles measured on the surface of *Hibiscus rosa-sinensis* pollen grain

**Figure 7:**
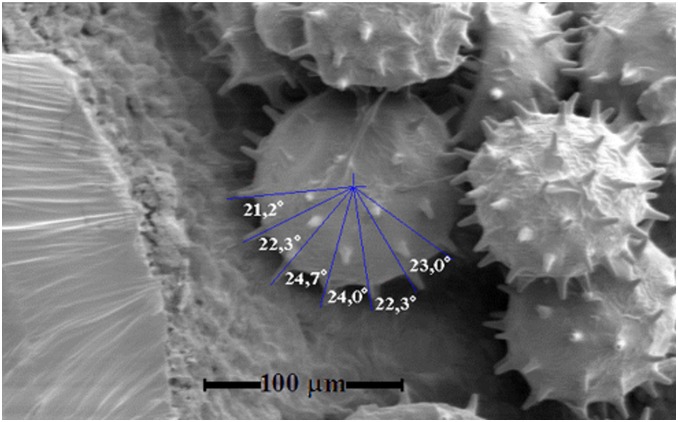
Microphotography of a pollen grain showing angle measurement of some of the spines on its surface.

In our study, we only consider experimental measurements from the length of the spines in the periphery of the grain since all other lengths are false because they are projections from a three dimensional space to a two dimensional space. Therefore errors during measurements arise in an automatic and inevitable way [9]. This is the case of the pollen grain. Although there are very good views regarding its morphology in all of the SEM pictures, the lengths of the angles between spines are obtained using computational calculations yielding a rough estimate of its magnitudes. Figure 7 shows an example of pollen grain with measurements of the angles between spines. We measured 100 angles, from them two thirds subtended by the hexagonal triangles, the rest by the pentagonal ones. The mean value is 23.7°±0.9° (standard deviation) for hexagons and 20.0°±0.6° for pentagons. This represents respectively a 1.8% and 0.2% difference from the theoretical values. So, experimental results are largely consistent with the theoretical predictions. Thus, the location of spines over the pollen surface is the same as that of the location of atoms in the buckminster C60-fullerene molecule. Thus, one of the first conclusions is that the pollen grain has the same symmetry group of this specific fullerene molecule. Another important taxonomy fact in pollen morphology is the number of spines. There are few reports about the number of spines in the pollen grain surface, and it depends on the species. In our case, the *Hibiscus rosa-sinensis* pollen having the same symmetry of the buckminster C60-fullerene molecule has 60 spines forming the spherical hexagons and pentagons. But in addition to this, it is necessary to consider that in the pollen grain the hexagons and pentagons are spine centered. It means that each of such figures have and additional point. If the number of spherical polygons in this specific fullerene molecule is 20 hexagons + 12 pentagons, then the number of polygons is 32. If each of them is centered, then there will be 32 point centers. Hence, the final account of spines in the pollen grain will be 60+32 = 92 spines. This number of spines had been reported in previous work [10], where the methodology to obtain it was a simple counting of pollen grain quadrant sectors in electron microscopy pictures.

The concept of spine index [7], or the ratio between the height and the width of the pollen spine, was measured in this work. The results match that of other works where such a concept is also considered [10]. But such a defined ratio could not be exact at all, since two similar spines would give the same spine index in two different stages of life. In addition this estimation leads to the impossibility of making a clear conclusion about the taxonomic identification of species within the same genera. So we introduce a new index called the D-spine index defined as the ratio between the radius of the pollen grain and a spine height on average.

All taken measurements are detailed in Table 1. From this study we can conclude that some mathematical parameters of the *Hibiscus rosa-sinensis* pollen morphology are not maintained throughout its development, such as diameter, pollen height, and spine index. All these changes are adaptation steps for an efficient pollination process. In this article, it is shown that the D-spine index shows a marked tendency when the grain pollen reaches its mature age. This parameter, introduced in our paper is more accurate for identifying the species of the same family, since we take into account a more general characteristic of the pollen, namely its diameter. Spine characterization is considered as a tool to discriminate the species within the family Malvaceae [7].

**Table 1:** Comparison of Spine index and D-spine-index in the three stages of pollen showing the variation as the grain reaches the end of its life span.

Pollenstage	Averagediameterof pollen(µm)	StandardDeviationdiameterof pollen(µm)	Averageheight ofpollen spine(µm)	StandardDeviationAverageheightof pollen spine(µm)	Average width ofpollen spine(µm)	Standard DeviationAverage width ofpollen spine(µm)	Spineindex	Error Spineindex	D-spineindex	ErrorD-spineindex
Young	95.5	3.5	11.0	1.4	7.0	0.4	1.6	0.3	4.3	0.02
Adult	89.4	7.4	10.9	1.4	6.5	0.4	1.7	0.3	4.1	0.03
Old	79.4	5.2	10.5	1.7	5.3	0.4	1.9	0.5	3.9	0.03

In the buckminster C60-fullerene molecule the idea of equidistant atoms gives an idea of chemical bonds of the same length and with a kind of electrical polarity that makes them separate in a symmetrical manner over the sphere’s area. Such bonds in the carbon atom are the hybrid bonds that exist in all of the exotic compounds the carbon atom can create. In the case of the pollen grain, the spines seem to have very important roles in the mechanical structure of the pollen as well as electrical properties [11] enabling the whole pollination process. From a mechanical and chemical point of view, the super-stability of this kind of structures has already been demonstrated [12], [13]. A distribution of spines equidistant from each other gives rise to repulsion forces due to electrical charges existing in the spines. The presence of electric charges has been reported in the Palynology literature. These are present in the pollen grain as well as in the insects paws which is the believed mechanism by the pollination process takes place. As it is well known, the pollination process is a very important process in maintaining the food chain of the whole ecosystem. Actually, around 60% of human food is from plants pollination [14]. The forces, facts, and other elements involved in the process are thoroughly studied. Our study had the goal of elucidating symmetrical concepts that lay in the pollen structure and said concepts could be generalized to other pollen species. In a future work, the hypothesis related to electrostatic charges and forces and metrical relationships involved in the pollination process of *Hibiscus rosa-sinensis* will be investigated.
